# Using the Implementation Leadership Scale (ILS) for nutrition incentive programs in the food retail context

**DOI:** 10.1186/s12966-026-01928-7

**Published:** 2026-05-06

**Authors:** James P. Marriott, Carmen Byker Shanks, Eric E. Calloway, Amy L. Yaroch, Joe Prickitt, Blanca Melendrez, Nancy Knauer, Bailey Houghtaling

**Affiliations:** 1 Center for Nutrition & Health Impact, Omaha, NE 68022 USA; 2 Department of Nutrition, University of North Carolina at Chapel Hill, Chapel Hill, NC 27599 USA; 3 Center for Community Health, University of California San Diego Altman Clinical & Translational Research Institute, 9452 Medical Center Drive, La Jolla, CA 92037 USA; 4 Mother’s Nutritional Center, 13635 Freeway Drive, Santa Fe Springs, CA 90670 USA; 5 Department of Human Nutrition, Foods, and Exercise, Virginia Tech, Blacksburg, VA 24061 USA

**Keywords:** Implementation science, Implementation Leadership Scale, Food retail, Nutrition incentives, Psychometric testing

## Abstract

**Background:**

The Implementation Leadership Scale (ILS) is widely used to measure implementation leadership for health innovations. While used often in behavioral health and other clinical settings, it remains untested in community public health contexts such as food retail. Healthy food retail strategies, including nutrition incentive programs, could benefit from measuring and subsequently strengthening leadership support to facilitate effective implementation. The objective of this study was to test the suitability of an adapted ILS to measure leadership support for a nutrition incentive program implemented in a brick-and-mortar food retail setting.

**Methods:**

As part of a larger evaluation, a multidisciplinary team of practitioners, evaluators, and food retail representatives created a modified version of the ILS suitable for the food retail context. Food retailer staff and management from one privately-owned grocery chain who participated in implementing a nutrition incentive program for Supplemental Nutrition Assistance Program (SNAP) shoppers in Southern California completed a survey that included the adapted ILS. Of the 522 survey respondents from the larger evaluation, 473 retailers including management and staff provided complete responses for the ILS. We assessed construct validity, internal consistency reliability, and measurement invariance using differential item functioning (DIF) analyses, Cronbach’s alpha, confirmatory factor analysis (CFA), and multiple-group CFA for the adapted ILS.

**Results:**

DIF analyses indicated minimal evidence of measurement bias. The CFA supported the original four-factor ILS structure, with excellent internal consistency for the knowledgeable, supportive, and perseverant subscales and fair consistency for the proactive subscale. Further, the demonstrated measurement invariance between management and staff highlights the robustness of the ILS and its potential for assessing alignment or discrepancies in perceptions of implementation leadership between management and staff within food retail organizations.

**Conclusions:**

Findings suggest that the adapted ILS is a valid and reliable tool for measuring implementation leadership support for nutrition incentive programs in a food retail setting in the US. Future research should examine the adapted ILS across diverse food retail environments and healthy food retail strategies to improve its generalizability and applicability.

**Supplementary Information:**

The online version contains supplementary material available at 10.1186/s12966-026-01928-7.

## Background

Effective leadership is key for facilitating the adoption, implementation, and sustainment of health innovations, according to multiple implementation science theories, models, and frameworks [1]. As one example, the Exploration, Preparation, Implementation, Sustainment (EPIS) framework identifies “Leadership” as a key contextual determinant that influences organizational actors’ ability and willingness to integrate health promoting innovations into standard practice [2, 3]. A key measure for implementation leadership in the field is the Implementation Leadership Scale (ILS), developed by Aarons et al. 2014, which is a 12-item pragmatic measure used to assess support for health innovations following four aspects of influential leadership – proactive, knowledgeable, supportive, and perseverant [4]. It has been extensively applied in behavioral health and other clinical settings throughout the United States (US) and other countries [5], and has garnered substantial empirical support, including extensive psychometric testing [4].

Despite these contributions, the broader field of implementation science contends with continued measurement gaps and instrumentation issues [5]. For example, a review found that nearly half of existing measures used to assess implementation of health-related practices lacked evidence of criterion validity [6]. Despite the importance of valid and reliable measures [7] and a growth in efforts in recent years [8], much work remains to understand the psychometric properties of implementation science measures used to assess implementation constructs and outcomes across a variety of contexts, including how these properties may change when existing measures are adapted [5]. Also, the application of implementation science methods and measures in community public health settings is typically understudied relative to clinical contexts [6–9]. While there are likely gradients of resource constraint between larger medical centers versus community-based healthcare organizations, community public health settings often lack payment and funding structures for health innovations and are also driven by different missions and values [6].

These key differences in organizational leadership, mission, and resources underscore the need to explore opportunities for contextually relevant measurement, including whether existing tools can be adapted for community public health settings. One key example is the food retail environment, which is increasingly recognized as an important leverage point for consumer health promotion, where organizational leadership can shape implementation success [10, 11]. An example of a growing U.S. healthy food retail intervention is nutrition incentive programs that aim to improve the affordability of fruits and vegetables among Supplemental Nutrition Assistance Program (SNAP) shoppers with low income through match-based incentives (e.g., spend $10, receive $10) at the point of sale [12, 13]. With SNAP being the largest federal food assistance program in the U.S [14], such nutrition incentive programs have the potential for wide-scale impact. However, food retail has historically been a challenging setting for health promotion and deserves more attention with respect to implementation research and practice [10, 11, 15].

As part of an implementation evaluation using the EPIS framework to understand brick-and-mortar retailers’ perceptions of implementing a nutrition incentive program [16], the ILS was adapted and used to understand leadership support within the food retail context. Given there are no available, validated implementation tools (to the authors’ knowledge) that have been used to assess implementation leadership for healthy food retail strategies like nutrition incentive programs, there was a need to examine whether the ILS functioned as intended when adapted for this context. Understanding the construct validity and measurement invariance of the ILS in the brick-and-mortar food retail setting can ensure accurate assessment of leadership behaviors and perceptions among managers and frontline staff and inform future efforts to support the implementation of consumer health promotion programs in these settings. Therefore, the specific aim of this study was to test the suitability of using an adapted ILS to measure leadership support for a nutrition incentive program implemented in a brick-and-mortar food retail setting by examining construct validity within this new context and measurement invariance between management and staff responses.

## Data and methods

### Adaptations to the implementation leadership scale

The ILS was created by Aarons et al. (2014) as a pragmatic and versatile tool, without specific reference to a health innovation, to assess four dimensions of leadership for successful implementation, including proactive, knowledgeable, supportive, and perseverant leadership [4]. While there is only one ILS, there are varied wording options that allow for both management and staff to respond to the ILS. For example, managers’ responses to the ILS reflect their perceptions of how effectively they have supported implementation, while staff responses reflect their perceptions of management’s support for implementation across the four leadership domains [4]. However, given that the ILS has been generated, tested, and primarily used within behavioral health and other clinical settings [4], slight adaptations were required to align the ILS with the brick-and-mortar food retail and nutrition incentive program context.

Therefore, in preparation for a larger evaluation study on the brick-and-mortar food retail implementation of a nutrition incentive program, a multidisciplinary team of practitioners, evaluators, and food retail representatives collaboratively modified the ILS wording through iterative team meetings and multiple rounds of revision for content validity [16]. In certain cases, terminology was adapted to make question language more accessible to food retailers following the advice of food retail representatives and practitioners. To provide a few examples, the phrase “facilitate implementation” was changed to “help carry out”, and “persevere” was changed to “persist”. Additionally, to align with the larger survey [16], response options for the ILS were altered from “Not at all; Slight extent; Moderate extent; Great extent; Very great extent” to “Completely disagree; Disagree; Neither agree nor disagree; Agree; Completely agree; Prefer not to answer; I don’t know and/or not applicable”. All adaptations to the ILS for use in the brick-and-mortar food retail context are available in Additional File 1. Mirroring the original ILS [4], slight wording variations were used for the adapted ILS for retail management versus staff respondents (Additional File 1).

### Nutrition incentive program

The nutrition incentive program was implemented in partnership with Mother’s Nutritional Center food retailers in California to provide a one-to-one match (up to $60 USD per month) to SNAP (also known as CalFresh in California) shoppers who purchased any fresh fruits and vegetables with their Electronic Benefits Transfer (EBT) cards [13]. Incentives were integrated directly onto EBT cards to be used for any SNAP-eligible food purchases at any SNAP-authorized retailer. See Prickitt et al., 2025 for more details on the nutrition incentive program [13].

Store managers and frontline staff played a central role in implementing the nutrition incentive program. Their responsibilities included ensuring accurate program delivery at the point of sale, stocking sufficient fresh fruits and vegetables to meet demand, processing incentive transactions through an upgraded point-of-sale system, and assisting customers with program navigation, incentive balances, and distinctions between the nutrition incentive program and other nutrition benefit programs. Staff also supported program marketing and participant engagement through store signage, reinforcement of training content, and customer education about the monthly incentive earning structure. Results on the implementation of the nutrition incentive program from food retailers’ perspectives, including ILS results, are presented separately [16]. Food retail management and staff both rated leadership support using the ILS highly; however, there were statistically significant lower values for staff ratings of management compared to management’s ratings of themselves [16].

### Sample and setting

The larger implementation evaluation surveyed 552 employees across 79 stores at a privately-owned grocery chain (Mother’s Nutritional Center) in Southern California [16]. All surveyed employees participated in the implementation of the nutrition incentive program in some capacity. Most respondents were female (76%) and identified as Hispanic, Latino/a, or Spanish (94%). On average, retailer staff were younger and employed for less time than retailer management (median age = 22 years vs. 31 years; 68.1% vs. 2.9% employed for less than 1 year) [16]. Germane to this analysis, retail employees included organizational- and store-level management (i.e., owner, directors, managers, 3rd persons [employees who mainly assist in managerial tasks], and crew leaders) and store-level staff (i.e., cashiers, produce attendants, and stockers). Only those with complete responses for all ILS items were included in the analytic dataset for the present study (*n* = 473; retailer management = 272; retailer staff = 201).

### Data analysis

Multiple analytic strategies were used to test measurement bias, construct validity, and reliability of the adapted ILS. First, differential item functioning (DIF) was assessed through the Cochran-Mantel-Haenszel procedure using the statistical software package jMetrik (Version 4.1.1). We conducted DIF for three demographic groups (age, gender, and Hispanic ethnicity). Hispanic ethnicity was chosen rather than a broader race/ethnicity grouping since 94% of survey respondents identified as Hispanic, Latino/a, or Spanish. Each demographic variable was transformed into binary reference and focal groups. Reference and focal groups were chosen based on the common practice that reference groups are considered to be socially advantaged over the focal groups [17]. For this study, we hypothesized the focal groups to be younger age (i.e., below the sample median age), Hispanic ethnicity, and non-male gender (i.e., female and non-binary/third gender) given the social and demographic characteristics of the study sample, whereas the reference groups were older age, non-Hispanic ethnicity, and male gender. For each ILS item across the demographic variables, we calculated standardized P-DIF statistics with 95% confidence intervals. Values around 0.0 indicate little DIF. The DIF categories (i.e., A, B or C) indicate low, moderate, or high levels of DIF, respectively. A positive sign for the B and C categories indicates that the item is biased toward the focal group, while a negative sign indicates favoring the reference group.

Next, a second-order confirmatory factor analysis (CFA) was conducted across the entire sample (*n* = 473) to confirm the previously established ILS factor structure (4). CFA is used as a test of construct validity by comparing how well the factor structure that underlies the scale matches the observed responses from the data [18]. To conduct the CFA, we used the lavaan package in R (R version 4.4.1) with the diagonally weighted least squares procedure due to the ordinal ILS variables and non-normal data distribution [19, 20]. We used root mean square error of approximation (RMSEA), standardized root mean square residual (SRMR), and the comparative fit index (CFI) to assess adequate model fit. Fit thresholds were established using cutoffs described by Hu and Bentler, 1999 (i.e., CFI ≥ 0.95, RMSEA ≤ 0.06, and SRMR ≤ 0.08 indicate adequate fit) [21]. Cronbach’s alpha was calculated to assess internal consistency for each ILS subscale and the overall scale.

Additionally, a multiple group CFA was performed to test measurement invariance between retailer management and staff responses to confirm whether management and staff mean ILS scores can be compared. This was done beyond the original ILS procedures as some differences in ILS values were found between management and staff in the implementation evaluation [16]. Therefore, the study team was interested in assessing whether ILS scores between the two groups could be meaningfully compared to aid in informing implementation strategies to bolster leadership support. The multiple group CFA was similarly conducted using the lavaan package with the diagonally weighted least squares procedure. Given missingness and low samples for responses in the completely disagree, disagree, and neither agree nor disagree categories across items, these options were collapsed to allow for model convergence. Due to similar issues with model convergence, this analysis included a first-order model only. Consistent with recommendations in the field and other literature on the topic, the benchmarks we used to determine whether measurement invariance was present in the sample were: ΔCFI ≥ − 0.01, ΔRMSEA ≤ 0.015, and ΔSRMR ≤ 0.030 [22, 23]. We assessed configural invariance and successively constrained the model to assess metric and scalar invariance. Demonstrating metric invariance (i.e., weak factorial invariance) is required to compare the relationships of the latent variables between retailer management and staff, while demonstrating scalar invariance (i.e., strong factorial invariance) is required to compare the means of the latent variables between retailer management and staff [24, 25].

## Results

### Differential item functioning

Standardized p-DIF statistics and DIF categories can be found in Additional File 2. Across all demographic groups, the DIF results indicated minimal evidence of measurement bias, with nearly all items demonstrating negligible DIF. One item related to removal of implementation obstacles indicated moderate DIF among retail management, favoring the older age group; however, given that the bias was only moderate and for one item, we conducted the CFA without making changes to the ILS structure.

### Confirmatory factor analysis and internal consistency

**Fig. 1:**
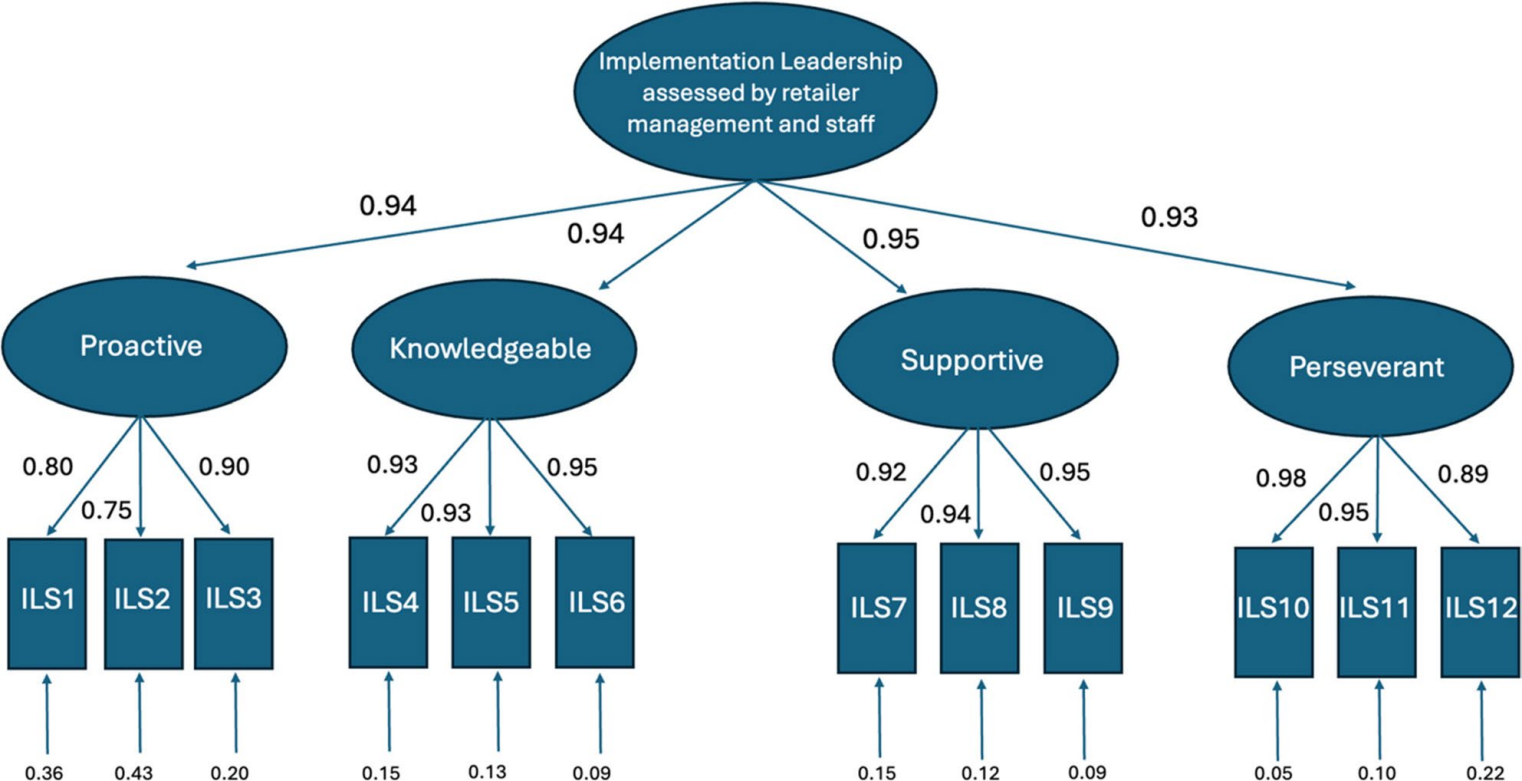
Second-order CFA factor loadings for the ILS

The CFA for the ILS among retailer management and staff demonstrated excellent fit for all metrics other than the chi-square test: CFI = 0.999; RMSEA = 0.048 (90% CI: 0.035–0.061); SRMR = 0.029; χ^2^ (50) = 105.252, *p* < 0.001. First-order and second-order standardized loadings and error variance are shown in Fig. 1. All factor loadings were statistically significant (*p* < 0.001). The Cronbach’s alpha for the 12-item ILS with combined retailer management and staff responses was 0.93, while the alpha values for the proactive, knowledgeable, supportive, and perseverant subscales were 0.68, 0.90, 0.91, and 0.90, respectively, indicating that the set of questions about proactive leadership demonstrated fair internal consistency reliability while the other subscales demonstrated excellent internal consistency [26]. Cronbach’s alpha values among retailer management only for the full 12-item scale, as well as proactive, knowledgeable, supportive, and perseverant subscales were 0.94, 0.76, 0.91, 0.93, and 0.90, while the values among retailer staff only were 0.92, 0.53, 0.89, 0.88, and 0.90 respectively. All values demonstrated acceptable internal consistency except for the proactive subscale among retailer staff, which demonstrated poor internal consistency.

### Multiple group confirmatory factor analysis

The results for the multiple group CFA are shown in Table 1. The first-order model for configural invariance demonstrated acceptable fit for all metrics other than the chi-square test: CFI = 0.999; RMSEA = 0.057 (90% CI: 0.043–0.071); SRMR = 0.039; χ2 (96) = 170.696, *p* < 0.001. After holding the first-order factor loadings equal in the metric invariance model, the model still demonstrated acceptable fit with CFI and SRMR, but not RMSEA and χ2, compared to the established fit thresholds: CFI = 0.998; RMSEA = 0.074 (90% CI: 0.062–0.086); SRMR = 0.044; χ2 (104) = 238.379, *p* < 0.001. The changes in fit indices are generally small and acceptable, although the change in RMSEA is slightly above the determined threshold: ΔCFI = -0.001; ΔRMSEA = 0.017; ΔSRMR = 0.005. Given the demonstrated support for metric invariance, we proceeded to further constrain the model to assess scalar invariance. The model demonstrated acceptable fit (χ2 (112) = 177.774, *p* < 0.001; CFI = 0.999; RMSEA = 0.050 (90% CI: 0.036–0.063); SRMR = 0.039) and acceptable changes in fit indices (ΔCFI = 0.001; ΔRMSEA = -0.024; ΔSRMR = -0.005). This indicates support for strong factorial invariance. Findings indicate that retail management and staff interpreted ILS items similarly, allowing for meaningful comparison of latent means between the two groups.

**Table 1:** Multiple group CFA fit statistics and comparisons for configural, metric, and scalar invariance models

Model	χ^2^	df	*p*	CFI	RMSEA	SRMR	Δ χ^2^	Δdf	ΔCFI	ΔRMSEA	ΔSRMR
Configural	170.696	96	< 0.001	0.999	0.057	0.039	-	-	-	-	-
*Retailer management*	*60.480*	*48*	*0.107*	*1.000*	*0.031*	*0.033*	-	-	-	-	*-*
*Retailer staff*	*110.216*	*48*	*< 0.001*	*0.999*	*0.081*	*0.048*	-	-	-	-	*-*
Metric^1^	238.379	104	< 0.001	0.998	0.074	0.044	67.683^3^	8	-0.001	0.017	0.005
Scalar^2^	177.774	112	< 0.001	0.999	0.050	0.039	60.605^3^	8	0.001	-0.024	-0.005

## Discussion

This study found the adapted ILS to be a generally valid and reliable tool for assessing leadership support for nutrition incentive programs in our study’s brick-and-mortar food retail context, which may have broader applicability for use among other healthy food retail strategies and in other food retail settings. These findings also help to extend the evidence base for leadership support measurement beyond traditional behavioral health and clinical settings (where much implementation research has been operationalized) to include community public health settings such as food retail [6–8]. Further, demonstration of measurement invariance between management and staff points to the robustness of the ILS and its potential for assessing alignment or discrepancies in perceptions of implementation leadership, which have been noted to have important implications on the implementation of evidence-based practices in mental health and school-based settings [27, 28]. Given we found evidence of some differences in perception in leadership support for the nutrition incentive program in the food retail context [16] results from the adapted ILS could be used to inform the need for and design of appropriate implementation strategies for leadership development [7] to support adoption, implementation, and sustainment.

The findings from this study largely align with those of the original ILS validation study by Aarons et al., 2014 [4] in that the adapted scale demonstrated strong overall internal consistency and construct validity. However, the results of this study do suggest a few opportunities to further test the suitability of the ILS in a variety of food retail settings. For example, it will be important to determine if the lower Cronbach’s alpha for the proactive subscale among retailer staff is specific to the one private, chain food retailer included in this study or food retail generally. It is possible that the items within this subscale, which focused on removing obstacles, developing a plan, and establishing clear standards, may be easier for management to answer, and staff may not have the requisite knowledge to respond consistently. Future users of the ILS within food retail may consider investigating this further to understand if these results hold among retailers with different business structures, workflows, and employee hierarchies before determining whether it is appropriate to remove this subscale for retail staff respondents. Additionally, more testing across different community public health innovation contexts, including other types of healthy food retail strategies (beyond nutrition incentives and perhaps those more applicable to other countries) and among other types of food retail settings (e.g., larger grocers, supercenters) will strengthen confidence in the ILS’s ability to be applied in different food retail formats.

There are several strengths to this study. It is the first to test the ILS in a healthy food retail context in the US, and findings may be applicable and translatable to other countries. Further, it is the first study to our knowledge that conducted a multiple group CFA using ILS data from both staff and management. The employment structure of the food retailer allowed us to survey sizable samples of food retail staff and management, providing a unique opportunity to directly compare staff and management scores. In addition, the ILS was adapted to suit the food retailer population in partnership with researchers, evaluators, and food retail representatives to ensure content validity [16], which is noted by the COnsensus-based Standards for the selection of health Measurement INstruments (COSMIN) as the most important measurement property [29].

However, there are limitations to this study. Survey respondents comprised a relatively homogeneous sample of food retailers (i.e., majority Latina) in Southern California, which may limit the external validity of these findings. Although we considered exploring convergent and discriminant validity, as assessed in the original scale development procedures [4], we were unable to do so given practical survey constraints. The primary goal of the holistic study was to evaluate a real-world program, and therefore we developed the survey in collaboration with study partners to meet the evaluation goals and minimize participant burden. Another limitation is that we observed ceiling effects with the ILS responses in that most respondents selected the highest response option (i.e., completely agree) for the items and few respondents selected the lower response options (i.e., completely disagree, disagree, and neither agree nor disagree). Consequently, we needed to collapse the lower response options to achieve model convergence for the multiple group CFA. Given that ceiling effects were not present in the initial scale validation [4] and food retailers in the present study indicated overwhelming support for both the program and its implementation [16], we believe that the lack of response variation is real and a result of retailers’ positive sentiments. For example, food retailers included in the evaluation study indicated excellent alignment between the innovation and the store’s mission, as well as robust implementation and preparation support from nutrition incentive program practitioners and higher-level food retail leadership. However, ideally future research in this area will utilize data with greater response variety. For example, application of the tool at various points of the implementation process may yield different findings (adoption versus preparation and implementation) [2, 3], whereas the ILS in this study was applied after several supports were provided to food retailers for nutrition incentive program implementation [16].

## Conclusions

The adapted ILS demonstrates strong construct validity and measurement invariance in a California-based food retail context, supporting its use for assessing leadership support for nutrition incentive program implementation. The scale’s robustness across management and staff respondents enables meaningful comparisons of perceived leadership engagement and alignment across organizational roles. Therefore, the adapted ILS could be used to inform opportunities to support the adoption, implementation, sustainment, and future scaling of nutrition incentive programs to improve consumers’ access to beneficial nutrition programming. Future efforts are needed to test the adapted ILS among a broader range of food retail contexts and healthy food retail strategies to strengthen generalizability and applicability in the US and beyond.

## Supplementary Information

Supplementary Material 1.

Supplementary Material 2.

## Data Availability

The datasets generated and/or analyzed during the current study are available from the corresponding author on reasonable request.

## Abbreviations

CFA: Confirmatory factor analysis
CFI: Comparative fit index
df: Degrees of freedom
DIF: Differential item functioning
ILS: Implementation Leadership Scale
RMSEA: Root mean square error of approximation
SNAP: Supplemental Nutrition Assistance Program
SRMR: Standardized root mean square residual
US: United States
χ^2^: Chi-square
