# Supplementary material for: Using the Implementation Leadership Scale (ILS) for nutrition incentive programs in the food retail context
Source: Int J Behav Nutr Phys Act. 2026 May 6;23:65. doi: 10.1186/s12966-026-01928-7 (PMC13321483; doi:10.1186/s12966-026-01928-7)
Supplement: Supplementary file 1 — Supplementary Material 1. [file 12966_2026_1928_MOESM1_ESM.docx]

**Using the Implementation Leadership Scale (ILS) for Nutrition Incentive Programs in the Food Retail Context: Supplemental Material**

**Retailer Leadership Version**

*Next, we would like to learn about your support for the CalFresh Fruit and Vegetable EBT Pilot Project as MNC leadership/management.*

*Please indicate your level of agreement with each statement.*

| 0 | 1 | 2 | 3 | 4 | - | - |
| --- | --- | --- | --- | --- | --- | --- |
| Completely disagree | Disagree | Neither agree nor disagree | Agree | Completely agree | Prefer not to answer | I don’t know and/or not applicable |

**Proactive**

1. I have provided staff with the tools and resources to help carry out the CalFresh Fruit and Vegetable EBT Pilot Project in MNC stores/my store.
2. I have removed obstacles to carrying out the CalFresh Fruit and Vegetable EBT Pilot Project in MNC stores/my store.
3. I have established clear standards for carrying out the CalFresh Fruit and Vegetable EBT Pilot Project in MNC stores/my store.

**Knowledgeable**

1. I am knowledgeable about the CalFresh Fruit and Vegetable EBT Pilot Project.
2. I can answer staff’s questions about the CalFresh Fruit and Vegetable EBT Pilot Project.
3. I know what I am talking about when it comes to the CalFresh Fruit and Vegetable EBT Pilot Project.

**Supportive**

1. I recognize and appreciate employee efforts toward successfully carrying out the CalFresh Fruit and Vegetable EBT Pilot Project.
2. I support employee efforts to learn more about the CalFresh Fruit and Vegetable EBT Pilot Project.
3. I support employee efforts to carry out the CalFresh Fruit and Vegetable EBT Pilot Project.

**Perseverant**

1. I persist through the ups and downs of carrying out the CalFresh Fruit and Vegetable EBT Pilot Project.
2. I continue through the challenges of carrying out the CalFresh Fruit and Vegetable EBT Pilot Project.
3. I respond to critical issues about carrying out the CalFresh Fruit and Vegetable EBT Pilot Project by openly and effectively addressing the problem(s).

**Retailer Staff Version**

*Next, we would like to learn about MNC store leadership/management support for the CalFresh Fruit and Vegetable EBT Pilot Project in your store from your perspective.*

*Please indicate your level of agreement with each statement.*

| 0 | 1 | 2 | 3 | 4 | - | - |
| --- | --- | --- | --- | --- | --- | --- |
| Completely disagree | Disagree | Neither agree nor disagree | Agree | Completely agree | Prefer not to answer | I don’t know and/or not applicable |

**Proactive**

1. MNC leadership/management has provided the tools and resources to help carry out the CalFresh Fruit and Vegetable EBT Pilot Project.
2. MNC leadership/management has removed obstacles to carrying out the CalFresh Fruit and Vegetable EBT Pilot Project.
3. MNC leadership/management has established clear store standards for carrying out the CalFresh Fruit and Vegetable EBT Pilot Project.

**Knowledgeable**

1. MNC leadership/management is knowledgeable about the CalFresh Fruit and Vegetable EBT Pilot Project.
2. MNC leadership/management can answer my questions about the CalFresh Fruit and Vegetable EBT Pilot Project.
3. MNC leadership/management knows what they are talking about when it comes to the CalFresh Fruit and Vegetable EBT Pilot Project.

**Supportive**

1. MNC leadership/management recognizes and appreciates employee efforts toward successfully carrying out the CalFresh Fruit and Vegetable EBT Pilot Project.
2. MNC leadership/management supports employee efforts to learn more about the CalFresh Fruit and Vegetable EBT Pilot Project.
3. MNC leadership/management supports employee efforts to carry out the CalFresh Fruit and Vegetable EBT Pilot Project.

**Perseverant**

1. MNC leadership/management persists through the ups and downs of carrying out the CalFresh Fruit and Vegetable EBT Pilot Project.
2. MNC leadership/management continues through the challenges of carrying out the CalFresh Fruit and Vegetable EBT Pilot Project.
3. MNC leadership/management responds to critical issues about carrying out the CalFresh Fruit and Vegetable EBT Pilot Project by openly and effectively addressing the problem(s).
