# Supplementary material for: Using the Implementation Leadership Scale (ILS) for nutrition incentive programs in the food retail context
Source: Int J Behav Nutr Phys Act. 2026 May 6;23:65. doi: 10.1186/s12966-026-01928-7 (PMC13321483; doi:10.1186/s12966-026-01928-7)
Supplement: Supplementary file 2 — Supplementary Material 2. [file 12966_2026_1928_MOESM2_ESM.docx]

**Using the Implementation Leadership Scale (ILS) for Nutrition Incentive Programs in the Food Retail Context: Supplemental Material**

Supplemental Table 1a. Differential Item Functioning Testing by Age for Retailer Management and Staff Respondents.^1^

| ILS Factor | Item | Group | sP-DIF (95% CI) | DIF Category |
| --- | --- | --- | --- | --- |
| Proactive | Provision of tools and resources | Management | -0.03 (-0.15, 0.09) | A |
|  |  | Staff | -0.07 (-0.31, 0.18) | A |
|  | Removal of implementation obstacles | Management | -0.29 (-0.53, -0.05) | B- |
|  |  | Staff | 0.01 (-0.31, 0.34) | A |
|  | Establishing clear standards | Management | -0.03 (-0.20, 0.14) | A |
|  |  | Staff | 0.06 (-0.08, 0.20) | A |
| Knowledgeable | Knowledgeable about the program | Management | 0.10 (-0.04, 0.25) | A |
|  |  | Staff | 0.00 (-0.14, 0.14) | A |
|  | Ability to answer questions | Management | 0.13 (0.01, 0.25) | A |
|  |  | Staff | -0.04 (-0.15, 0.07) | A |
|  | Knowing what they are talking about | Management | 0.10 (0.00, 0.19) | A |
|  |  | Staff | -0.04 (-0.16, 0.09) | A |
| Supportive | Recognizing and appreciating employee efforts | Management | -0.05 (-0.17, 0.06) | A |
|  |  | Staff | -0.08 (-0.22, 0.06) | A |
|  | Supporting employee efforts to learn more about the program | Management | -0.08 (-0.20, 0.04) | A |
|  |  | Staff | -0.06 (-0.19, 0.07) | A |
|  | Supporting employee efforts to carry out the program | Management | -0.02 (-0.11, 0.08) | A |
|  |  | Staff | -0.02 (-0.15, 0.10) | A |
| Perseverant | Persisting through the ups and downs | Management | 0.04 (-0.07, 0.14) | A |
|  |  | Staff | 0.09 (-0.04, 0.22) | A |
|  | Continuing through the challenges | Management | 0.13 (0.01, 0.26) | A |
|  |  | Staff | 0.09 (-0.04, 0.22) | A |
|  | Responding to critical issues | Management | -0.00 (-0.12, 0.11) | A |
|  |  | Staff | 0.05 (-0.09, 0.19) | A |

^1^Focal group = younger age, n = 225; reference group = older age, n = 227. Younger vs. older age was determined by splitting the respondents by median age.

Supplemental Table 1b. Differential Item Functioning Testing by Gender for Retailer Management and Staff Respondents.^1^

| ILS Factor | Item | Group | sP-DIF (95% CI) | DIF Category |
| --- | --- | --- | --- | --- |
| Proactive | Provision of tools and resources | Management | 0.07 (-0.05, 0.20) | A |
|  |  | Staff | 0.02 (-0.14, 0.17) | A |
|  | Removal of implementation obstacles | Management | 0.11 (-0.03, 0.25) | A |
|  |  | Staff | -0.16 (-0.33, 0.01) | A |
|  | Establishing clear standards | Management | -0.01 (-0.09, 0.07) | A |
|  |  | Staff | -0.01 (-0.13, 0.10) | A |
| Knowledgeable | Knowledgeable about the program | Management | 0.03 (-0.06, 0.12) | A |
|  |  | Staff | -0.06 (-0.17, 0.05) | A |
|  | Ability to answer questions | Management | 0.00 (-0.07, 0.08) | A |
|  |  | Staff | -0.04 (-0.12, 0.05) | A |
|  | Knowing what they are talking about | Management | -0.02 (-0.09, 0.04) | A |
|  |  | Staff | 0.05 (-0.03, 0.13) | A |
| Supportive | Recognizing and appreciating employee efforts | Management | 0.05 (-0.02, 0.11) | A |
|  |  | Staff | 0.02 (-0.06, 0.11) | A |
|  | Supporting employee efforts to learn more about the program | Management | 0.01 (-0.06, 0.08) | A |
|  |  | Staff | 0.09 (0.02, 0.17) | A |
|  | Supporting employee efforts to carry out the program | Management | -0.04 (-0.13, 0.04) | A |
|  |  | Staff | 0.02 (-0.06, 0.09) | A |
| Perseverant | Persisting through the ups and downs | Management | -0.06 (-0.15, 0.02) | A |
|  |  | Staff | -0.01 (-0.09, 0.07) | A |
|  | Continuing through the challenges | Management | -0.09 (-0.16, -0.01) | A |
|  |  | Staff | -0.01 (-0.09, 0.06) | A |
|  | Responding to critical issues | Management | -0.05 (-0.14, 0.04) | A |
|  |  | Staff | 0.10 (0.01, 0.18) | A |

^1^Focal group = non-male (i.e., female and non-binary/third gender), n = 362; reference group = male, n = 110.

Supplemental Table 1c. Differential Item Functioning Testing by Hispanic Ethnicity for Retailer Management and Staff Respondents.^1^

| ILS Factor | Item | Group | sP-DIF (95% CI) | DIF Category |
| --- | --- | --- | --- | --- |
| Proactive | Provision of tools and resources | Management | -0.02 (-0.16, 0.11) | A |
|  |  | Staff | -0.05 (-0.20, 0.10) | A |
|  | Removal of implementation obstacles | Management | -0.15 (-0.36, 0.06) | A |
|  |  | Staff | -0.08 (-0.28, 0.13) | A |
|  | Establishing clear standards | Management | 0.20 (0.06, 0.34) | A |
|  |  | Staff | 0.06 (-0.02, 0.13) | A |
| Knowledgeable | Knowledgeable about the program | Management | 0.00 (-0.07, 0.08) | A |
|  |  | Staff | -0.02 (-0.07, 0.02) | A |
|  | Ability to answer questions | Management | 0.09 (-0.01, 0.19) | A |
|  |  | Staff | -0.01 (-0.06, 0.04) | A |
|  | Knowing what they are talking about | Management | 0.02 (-0.03, 0.07) | A |
|  |  | Staff | -0.03 (-0.11, 0.05) | A |
| Supportive | Recognizing and appreciating employee efforts | Management | -0.06 (-0.13, 0.02) | A |
|  |  | Staff | 0.07 (-0.01, 0.15) | A |
|  | Supporting employee efforts to learn more about the program | Management | -0.01 (-0.10, 0.07) | A |
|  |  | Staff | 0.03 (-0.04, 0.11) | A |
|  | Supporting employee efforts to carry out the program | Management | 0.01 (-0.07, 0.09) | A |
|  |  | Staff | 0.03 (-0.04, 0.11) | A |
| Perseverant | Persisting through the ups and downs | Management | -0.07 (-0.17, 0.09) | A |
|  |  | Staff | -0.03 (-0.11, 0.04) | A |
|  | Continuing through the challenges | Management | -0.06 (-0.15, 0.03) | A |
|  |  | Staff | 0.09 (0.01, 0.17) | A |
|  | Responding to critical issues | Management | 0.05 (-0.06, 0.16) | A |
|  |  | Staff | -0.01 (-0.10, 0.07) | A |

^1^Focal group = Hispanic, n = 448; reference group = non-Hispanic, n = 21.
